# Temporal progression of PARP activity in the *Prph2* mutant *rd2* mouse: Neuroprotective effects of the PARP inhibitor PJ34

**DOI:** 10.1371/journal.pone.0181374

**Published:** 2017-07-19

**Authors:** Ayse Sahaboglu, Alaa Sharif, Lili Feng, Enver Secer, Eberhart Zrenner, François Paquet-Durand

**Affiliations:** 1 Institute for Ophthalmic Research, University of Tuebingen, Tuebingen, Germany; 2 Graduate Training Center of Neuroscience, Tuebingen, Germany; 3 Department of Medical Genetics, Erciyes University, Kayseri, Turkey; 4 Centre for Integrative Neuroscience (CIN), University of Tuebingen, Tuebingen, Germany; University of PECS Medical School, HUNGARY

## Abstract

Peripherin (peripherin/*rds*) is a membrane-associated protein that plays a critical role in the morphogenesis of rod and cone photoreceptor outer segments. Mutations in the corresponding *PRPH2* gene cause different types of retinal dystrophies characterized by a loss of photoreceptors. Over activation of poly-ADP-ribose polymerase (PARP) was previously shown to be involved in different animal models for hereditary retinal dystrophies. This includes the *rd2* mouse, which suffers from a human homologous mutation in the *PRPH2* gene. In the present study, we show that increased retinal PARP activity and poly-ADP-ribosylation of proteins occurs before the peak of *rd2* photoreceptor degeneration. Inhibition of PARP activity with the well-characterized PARP inhibitor PJ34 decreased the levels of poly-ADP-ribosylation and photoreceptor cell death. These results suggest a causal involvement of PARP in photoreceptor degeneration caused by peripherin mutations and highlight the possibility to use PARP inhibition for the mutation-independent treatment of hereditary retinal dystrophies.

## Introduction

The photoreceptors of the retina are an exceptional type of neuron, highly specialized to their unique task of transforming photons of light into electrochemical messages. While rod photoreceptors are adapted to detect very low light levels, cone photoreceptors can perceive bright light with different spectral sensitivities providing for colour vision and—in the human situation—for high resolution. The signal conversion takes place in the photoreceptor outer segments (OS) where stacks of membranous disks harbour the key components of the phototransduction cascade. The edge of each OS disk is attached to the extracellular membrane by a glycoprotein called peripherin/rds [1]. Mutations in the corresponding *PRPH2* gene disrupt OS architecture and are the cause of blinding hereditary retinal degeneration. Depending on the exact nature of the mutation (*e*.*g*. missense, frame-shift, premature stop) the disease phenotype may vary considerably and may be attributed to several rare retinopathies including Retinitis Pigmentosa (RP), Leber´s congenital Amaurosis (LCA), or cone dystrophy [2,3]. Overall, these diseases are characterized by a very marked genetic heterogeneity [4] with causal mutations in over 250 different genes, including in *PRPH2* (https://sph.uth.edu/RetNet; information retrieved March 2017).

During normal rodent photoreceptor differentiation and maturation the photoreceptor OSs start to grow out at around post-natal day (P) 10 and reach full length at around P30 [5]. The peripherin/rds mutant *rd2* mouse (*PRPH2* gene mutation) shows essentially no outer segment formation and slow and progressive photoreceptor degeneration [5–7]. Due to the failure to develop photoreceptor OSs the electroretinographic response never fully develops and progressively diminishes with time until it becomes virtually extinguished by 12 months of age [8]. The *rd2* mouse phenotype thus corresponds to the human patient situation, making the *rd2* mouse a relevant model for pathophysiological and therapeutic studies.

Recently, the enzyme PARP was found to be strongly activated at the peak of photoreceptor degeneration in ten different mouse and rat models for hereditary retinal degeneration, including also the *rd2* mouse [9]. PARP enzymes catalyse mono- and poly-ADP-ribosylation of proteins, an important post-translational modification which is implicated in a wide range of cellular processes [10]. These include DNA repair and maintenance of genomic stability, transcriptional regulation, energy metabolism, and cell death [11,12]. In recent years, the involvement of PARP in a variety of diseases has led to the development of a number of highly specific PARP inhibitors, many of which have been tested clinically [13].

In the present study, we have assessed and compared the progression of both *rd2* retinal degeneration and PARP activity and found them to closely correlate, with the peak of PARP activity preceding cell death by about two days. Furthermore, the well-characterized PARP inhibitor PJ34 had a marked protective effect in *rd2* retinal explant cultures, reducing photoreceptor cell death and restoring in part the normal architecture of photoreceptor OSs. Our study establishes PARP activity as an important contributor to *rd2* photoreceptor cell death and provides a novel approach for the prevention of both rod and cone photoreceptor loss in *rd2* retinal degeneration.

## Material and methods

### Experimental animals

**Fig 1:**
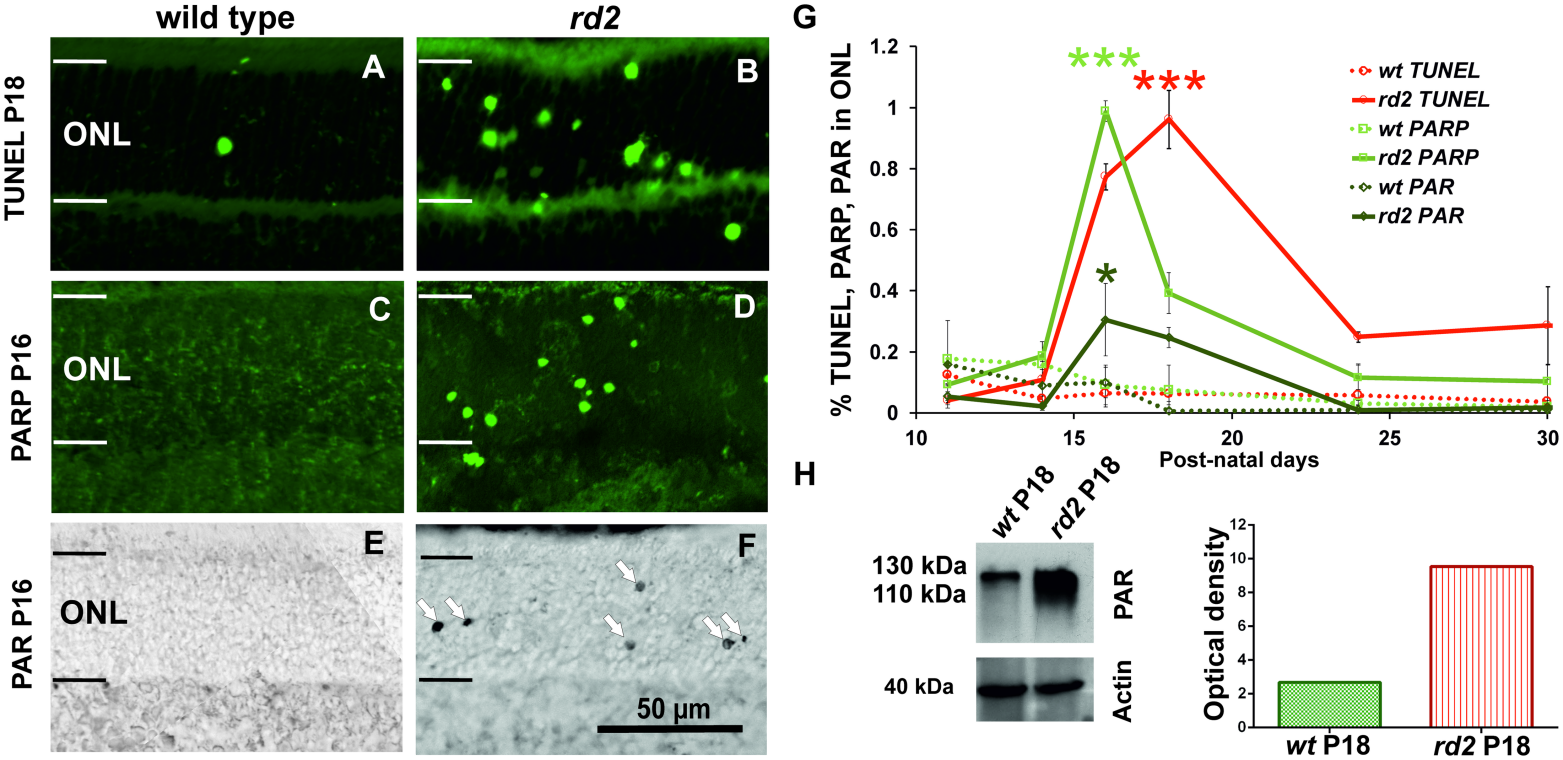
Progression of cell death and PARP activity in the early post-natal rd2 retina. The TUNEL assay for dying cells showed significantly increased numbers of positive cells between P14 and P24, and peaked at P18 in *rd2* retinae (A,B,G). The quantification of PARP activity positive cells over time identified significantly higher numbers of positive cells at P16 and P18 in *rd2* ONL (C,D,G). Similar to PARP activity, immunohistochemical analysis of PARylated proteins revealed significantly increased numbers of PAR positive cells at P16 and P18 in *rd2* retina (E,F,G). Remarkably, while PARP activity and PARylation of proteins peaked at P16, the peak of cell death occurred only at P18. Western blot analysis confirmed increased levels of PARylated proteins in *rd2* retina at P18. The images shown in A-F are representative for observations on at least three different specimens for each genotype. Data shown in G is based on marker quantifcations in three different wt and *rd2* animals per time-point; note that the wt datasets were in part published previously in [9,20].

C3H wild-type (wt) and C3H *rd2* animals [14] were housed under standard white cyclic lighting and had free access to food and water. All procedures were performed in accordance with the ARVO statement for the use of animals in ophthalmic and visual research and were approved by the Tübingen University committee on animal protection (Einrichtung für Tierschutz, Tierärztlicher Dienst und Labortierkunde directed by Dr. Franz Iglauer; Reg. dates: 11/03/2011, 08/12/2015). Animals were killed with CO_2_ and their eyes were enucleated. A total of 18 different *rd2* mice were used for *in vivo* studies (three per time point, *i*.*e*. post-natal days 11, 14, 16, 18, 24, 30) (Fig 1), with further six wild-type animals used for comparisons.

### Retinal explant cultures

**Fig 2:**
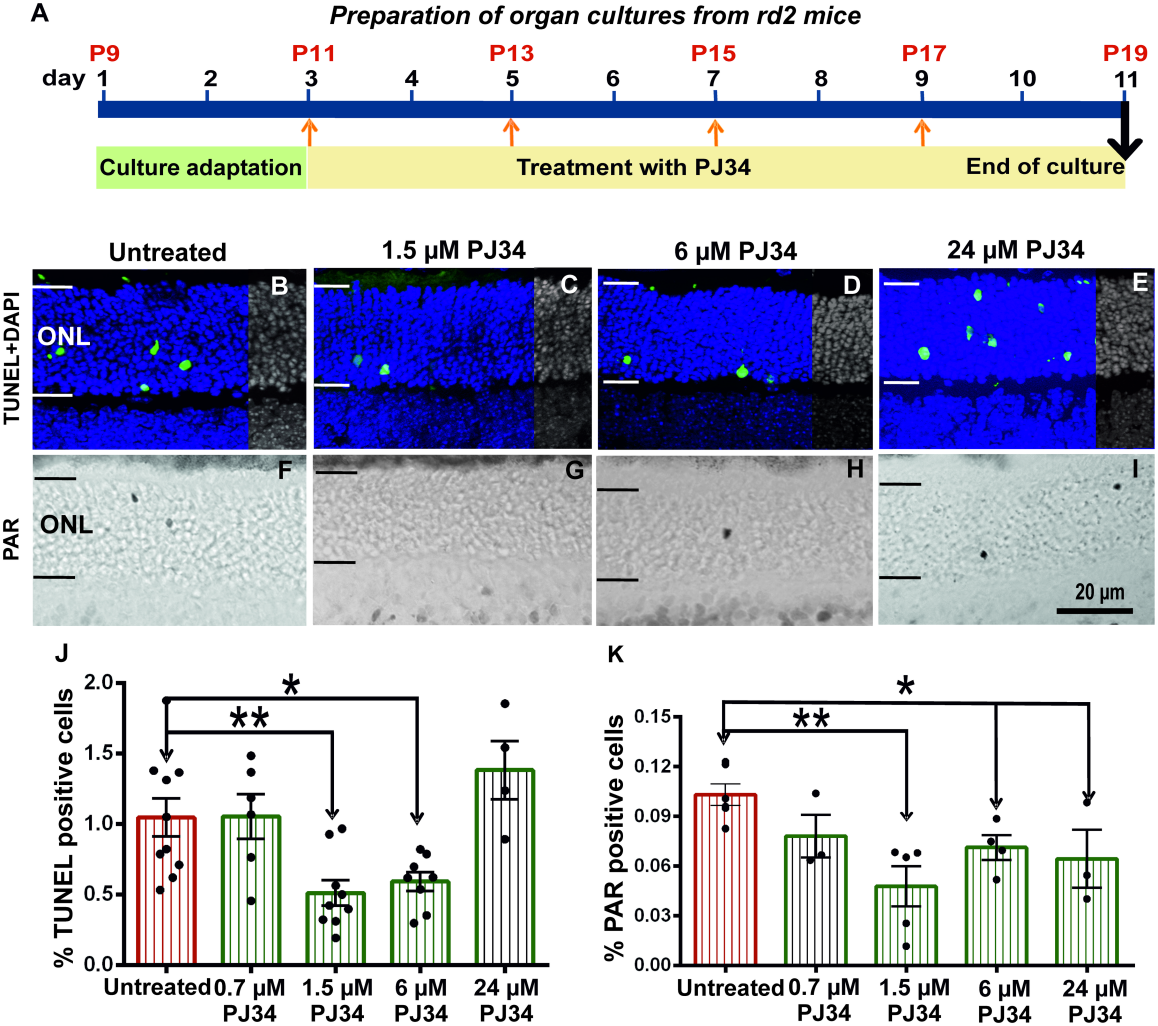
Treatment schedule and TUNEL, PARylation in rd2 retinal explants. Culture started at P9, after 2 days culture adaptation period *rd2* retinae treated with different concentrations of PJ34 between P11-P19 for 8 days. Culture experiments terminated at P19 (A). *rd2* retinae were treated with different concentrations of PJ34 and stained for dying cells using the TUNEL assay and PAR accumulation using immunohistochemistry (B-I). The number of TUNEL positive cells was significantly decreased in 1.5 μM and 6 μM PJ34 treated groups, while there were no differences in 0.7 μM and 24 μM PJ34 treated groups (B-E, J). Similarly, the numbers of ONL cells showing PAR accumulation were significantly lower in the explants that had been treated with PJ34 concentrations of 1.5 μM and higher (F-I, K). The images shown are representative for observations for at least 6 different specimens for each genotype.

Organotypic retinal cultures from *rd2* mice were prepared as previously described [15,16]. In brief, animals were sacrificed at post-natal day (P) 9, their eyes enucleated, and incubated for 15 min with 0.12% proteinase K (ICN Biomedicals Inc., OH, USA; 193504). The activity of proteinase K was blocked by 10% foetal calf serum (FCS) and rinsing with serum free medium. Cornea, sclera, lens and choroid were removed, only retina remained together with the RPE. Then the eye cup—including both retina and RPE—was cut into a clover-leaf-like shape and transferred to a culture membrane insert (Millipore, Carrigtwohill, Cork, Ireland; PIHA03050). The inserts were placed in six-well culture plates with R16 medium and supplements [17]. Cultures were incubated at 37°C in a humidified incubator with 5% CO_2_ between P9 from P19 for 10 days, and the culture medium was changed every two days. For the first two days (P9-P11) the cultures were left without treatment, and then treated for 8 days (P11-P19) with PJ34 (Sigma, Munich, Germany; Z0878), at concentrations ranging from 0.7–24 μM (Fig 2). PJ34 was dissolved in dimethyl sulfoxide (DMSO; Sigma; D2650) and diluted in R16 medium with supplements. The same concentrations of DMSO were added to the controls. 18 *rd2* mice (= 36 retinal explants) were used for culture experiments and assigned to the different treatment groups.

### Tissue preparation

Eyecups or retinal explant cultures were immersion fixed for 1h at room temperature (RT) in 4% paraformaldehyde (PFA) (Polysciences, Warrington PA, USA) in 0.1 M phosphate buffer (PB, pH 7.4) containing 0.2 M sucrose. After washing in PBS, eyes were cryoprotected by immersion in graded sucrose (10%, 20%, 30%) in PBS. Tissues were then embedded in a tissue-freezing medium (Jung, Leica Instruments, Heidelberg, Germany). Vertical °sections (12 μm) were cut on a Leica CM3050S Microtome (Leica Biosystems, Wetzlar, Germany), air dried at 37°C for 1h, and stored at -20°C until use. Frozen sections from fixed tissue were air dried for 30–60 min at 37°C.

### TUNEL assay and immunofluorescence

The terminal deoxynucleotidyl transferase dUTP nick end labelling (TUNEL) assay was performed on cryosections from *in vivo* and treated/untreated *rd2* retinae, using an *in situ* cell death detection kit conjugated with fluorescein isothiocyanate (Roche Diagnostics, Mannheim, Germany). For controls, terminal deoxynucleotidyl transferase enzyme was either omitted from the labelling solution (negative control), or sections were pre-treated for 30 min with DNAse I (Roche, 3U/ml) in 50 mM Tris-HCl, pH 7.5, 1 mg/ml BSA to induce DNA strand breaks (positive control). Negative control gave no staining at all, while positive control stained all nuclei in all layers of the retina.

For immunofluorescence (IF) sections were rinsed in PBS and pre-incubated for 1h at RT in blocking solution containing 10% normal goat serum, 1% bovine serum albumin (BSA), and 0.1% Triton X in PBS. Rhodopsin (Merck Millipore, Darmstadt, Germany; MAB5316) were diluted in blocking solution overnight at 4°C. Subsequently, sections were rinsed in PBS and incubated with Alexafluor-488 conjugated secondary antibody (Invitrogen; dilution 1:250–1:750). Sections were washed in PBS and mounted in Vectashield mounting medium with DAPI (Vector, Burlingame, CA, USA).

### PARP activity assay

Eyes from *wt* and *rd2* mice were enucleated, frozen immediately on dry ice (-72°C), followed by cryosectioning. A biotin-avidin blocking kit (Vector) was used to block endogenous biotin and to reduce background. After incubation with PARP reaction mixture (10 mM MgCl_2_, 1 mM dithiothreitol, 5 μM biotinylated NAD (Trevigen, Gaithersburg, MD, USA) in 100 mM Tris buffer with 0.2% Triton X100, pH 8.0) for 2.5 h at 37°C, the sections were washed 3 times with PBS (5 min). The biotin incorporated by PARP activity was then detected by fluorescently labelled avidin (1:800 in PBS, 1h at RT). After three washes in PBS (5 min), the sections were mounted in Vectashield (Vector). For controls, biotinylated-NAD^+^ was omitted from the reaction mixture resulting in absence of detectable reaction product.

### PAR immunohistochemistry

Poly ADP-ribose (PAR) immunohistochemistry was performed with sections from *in vivo* and *in vitro rd2* mice and wt. Sections were air dried 30–60 min at 37°C and washed with PBS for 10 min. Non-specific background reduced by quenching solution which included 30% H_2_O_2_, MeOH, 0,1% PBST. After that, sections were blocked with 10% normal goat serum in 0.1% PBST for 1h at RT and incubated with PAR antibody (PAR 10H, Alexis, dilution 1:200) for overnight at 4°C. Biotinylated secondary antibody (Vector; dilution 1:150) was diluted in 10% normal goat serum in 0.1% PBST and the sections were incubated for 1h at RT. After washing, the slides were incubated in Vectastain Elite ABC kit (Vector) for 1h at RT. The colour reaction was produced with 3,3'-diaminobenzidine (DAB) solution containing 20% glucose, 0.4% NH^4^Cl, 1% nickel ammonium sulphate, 40 mg DAB, 40μl glucose oxidase. After DAB incubation, slides were washed with PBS and covered by Aquatex (Merck).

### Western blot

Retinal tissue from wt (five animals) and *rd2* mice (five animals) were homogenized in buffer as described below with a Precellys 24 homogenizer (Bertin Technologies, France) or a manual homogenizer (glass to glass). Bradford assay was used for determination of protein concentration. For separation of proteins, SDS-PAGE 12% gradient gel was used and 30 μg protein was loaded per well. Subsequently, the proteins were transferred to PVDF membrane (GE Healthcare, UK). Membranes were blocked in Roti Block (Roth, Karlsruhe, Germany) blocking buffer for 2h at RT. Membranes were incubated with primary antibodies against PAR (10H, Enzo, Lörrach, Germany) actin (Sigma-Aldrich; A2668, dilution 1:1000) at a dilution of 1:1000 in buffer containing PBST and 5% dried milk (Roth) overnight at 4°C. Membranes were washed with PBST and incubated with horseradish peroxidase conjugated secondary antibody (GE Healthcare) detection system was used as a membrane developer. Films were scanned and quantified using ImageJ 1.48v (National Institutes of Health, Washington, USA).

### Quantification and statistics

Retinal tissue sections from *in vivo* and *in vitro* explant preparations were viewed under a Axio Imager Z1 ApoTome microscope, AxioCam MRm camera and AxioVision 4.7 software (all from Zeiss, Jena, Germany) in Z-stack and mosaic mode at 20X magnification. For quantitative analysis, positive cells in the entire ONL of four cross-sections per culture were counted manually. The percentage of positive cells was calculated by dividing the absolute number of positive cells by the total number of ONL cells, which was assessed by dividing ONL area by the size of a photoreceptor nucleus (18 μm^2^), as measured via DAPI staining.

For the quantification of OS length these were measured on retinal micrographs using Axiovision software (Zeiss). Ten measurements (1 every ≈ 40 μm) were obtained on 3–4 images for each retinal explant culture. Measurements were performed near the optic nerve, at a distance of approx. 200 μm; three different animals/explant cultures for each genotype/condition were analysed.

Statistical analysis was performed using GraphPad Prism 6 software (GraphPad software, La Jolla, CA, USA). For multiple comparisons (*i*.*e*. different concentrations of PJ34) the Dunn´s multiple comparisons test was used. For one-to-one comparisons (*e*.*g*. TUNEL, OS length) the unpaired Student’s t-test was used.

## Results

In a previous study [9], we had found that the peak of *rd2* photoreceptor degeneration was at P18, as assessed by quantification of dying cells, TUNEL assay in the outer nuclear layer (ONL). At this time-point there was a significant and strong elevation in the numbers of photoreceptors showing high PARP activity as well as accumulation of PAR. To further assess the extent and temporal pattern of this correlation, we first investigated PARP activity and PAR accumulation on serial retinal sections taken between P11 and P30.

### PARP activity correlates with rd2 photoreceptor cell death

Between P11 and P30, the ONL of wild-type (wt) retina showed only low amounts of developmental cell death as evidenced by the TUNEL assay, in line with earlier studies on retinal development [18]. By comparison, the numbers of dying TUNEL positive cells in *rd2* ONL were significantly elevated at P18 (Fig 1A and 1B; quantification in G; rd2: 0.96% ± 0.1 SEM, n = 3; wt: 0.06% ± 0.02 SEM, n = 3; p<0.0001). Similarly, wt ONL was essentially devoid of PARP activity and signs of PAR accumulation (P16 wt PARP activity: 0.09% ± 0.05 SEM, n = 3; P16 wt PAR: 0.09% ± 0.06 SEM, n = 3) while numerous *rd2* photoreceptors were positive for both (Fig 1D and 1F; P16 *rd2* PARP activity: 0.98% ± 0.03 SEM, n = 3, p<0.0001; P16 *rd2* PAR: 0.30% ± 0.11 SEM, n = 3, p<0.05). When the progression of cell death (TUNEL assay), PARP activity, and cellular PAR accumulation was plotted over time a strong correlation between these parameters was evident (Fig 1G). Remarkably, the peaks of PARP activity and PAR accumulation preceded the TUNEL peak by approximately two days, indicating that PARP activation could be a cause of cell death rather than a consequence.

The strong PAR accumulation in individual photoreceptor cells was confirmed by western blot (WB) analysis of whole retina tissue samples. When compared to wild-type retina, an increased tissue PAR accumulation was evident already at P14 (not shown). At P18 increased PAR levels were especially prominent around a 116 kDa band that corresponded to the molecular weight of PARP itself, indicating a likely auto-PARylation (Fig 1H).

### PARP inhibition delays rd2 photoreceptor degeneration

To study the neuroprotective effect of PJ34, the TUNEL assay was performed on *rd2* retinal cultures treated with different concentrations of PJ34 (0.7 μM, 1.5 μM, 6 μM, 24 μM; Fig 2A–2D). Comparison of PJ34 treated groups with untreated control showed no difference for 0.7 μM PJ34 treatment (untreated: 1.05 ± 0.1 SEM, n = 10, treated: 1.05 ± 0.2 SEM, n = 6; p = 0.969) and a significant difference for the 1.5 μM and 6 μM PJ34 treated groups (1.5 μM: 0.51 ± 0.1 SEM, n = 9, p = 0.005; 6 μM: 0.59 ± 0.1 SEM, n = 8; p = 0.013). In the 24 μM PJ34 group the numbers of dying cells were increased, however this effect was not statistically significant (treated: 1.38 ± 0.2 SEM, n = 4; Fig 2I).

### PARP inhibition decreases PARylation in rd2 retinal cultures

The effectiveness of PARP inhibition was analyzed by staining for PARylated proteins in photoreceptors. The quantification of PAR positive cells in ONL indicated not significant difference for 0.7 μM PJ34 treated group (treated: 0.08 ± 0.01 SEM, n = 3; untreated: 0.10 ± 0.01 SEM, n = 6, p = 0.090; Fig 2J). There was a significant decrease of PAR positivity for the 1.5 μM, 6 μM and 24 μM PJ34 treated groups (1.5 μM: 0.05 ± 0.0 SEM, n = 5, p = 0.002; 6 μM: 0.07 ± 0.01 SEM, n = 6, p = 0.013; 24 μM: 0.06 ± 0.0 SEM, n = 3, p = 0.035; Fig 2E–2H and 2J).

In summary, the *in vitro* analysis showed a neuroprotective effect for PJ34 treatment at concentrations of 1.5 μM and 6 μM. This result in turn suggested that PARP inhibition protected photoreceptors which have rds mutation.

### PARP inhibition improves rhodopsin localisation in rd2 outer segments

**Fig 3:**
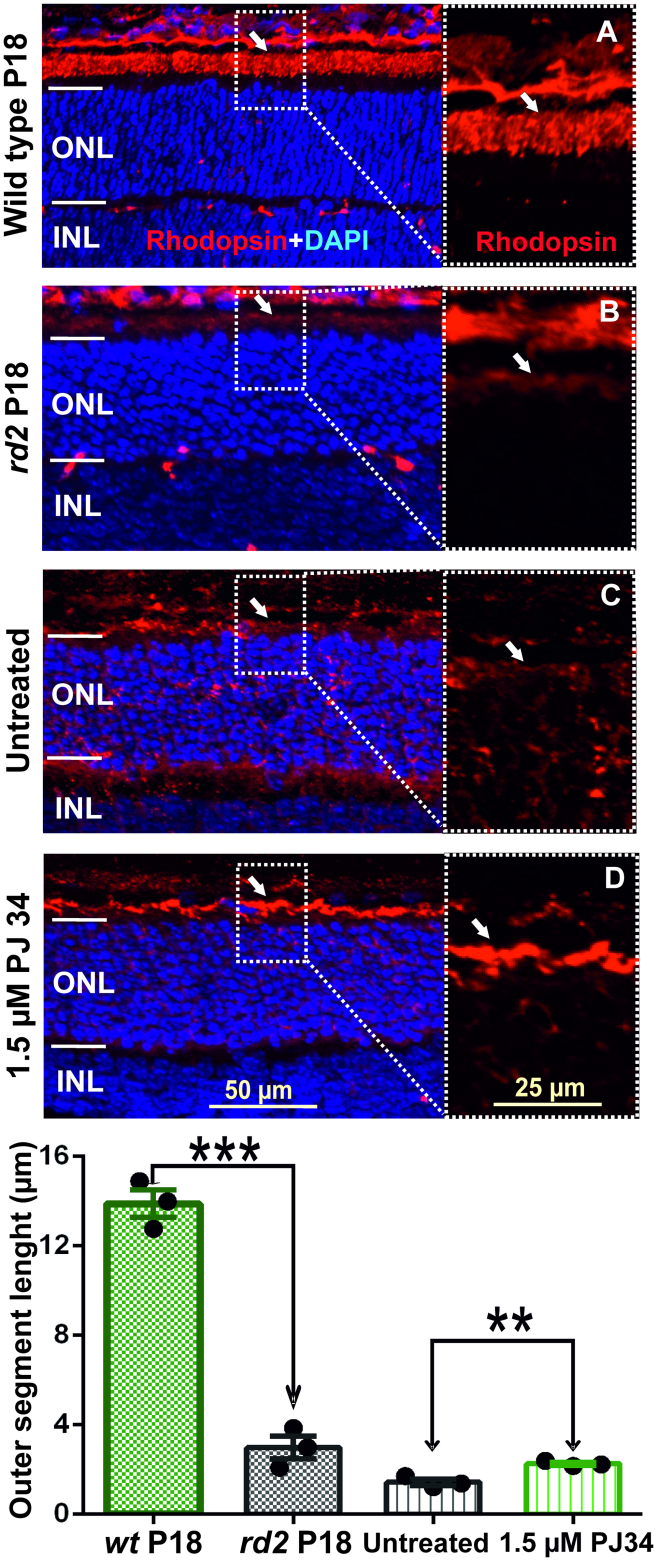
PJ34 treatment partially restores rhodopsin expression in rd2 photoreceptors. Immunostaining for rhodopsin showed a decreased expression level and dramatically shortened outer segments in P18 *rd2* retina, when compared with age-matched *wt* (A, B). Rhodopsin expression and OS length appeared to increase in *rd2* cultures that were treated with 1.5 μM PJ34 (C, D). The images shown are representative for observations on at least three different specimens for each genotype/treatment condition.

To test whether and how PARP inhibition would affect rod photoreceptor morphology we performed an immunostaining for rhodopsin. At P18, wild-type retina illustrated the normal development of rod outer segments (OS), characterized by strong rhodopsin immunoreactivity. In *rd2* rod OS rhodopsin expression was decreased and significantly different than *wt* (Fig 3A and 3B), in line with previous reports [19]. Likewise, *rd2* explant cultures showed a low expression of rhodopsin at P19 *in vitro*, an age corresponding approximately to P18 *in vivo*. In comparison, *rd2* explant cultures treated with 1.5 μM PJ34 showed significant OS growth and pronounced rhodopsin immunoreactivity (Fig 3C and 3D), indicating that the treatment had in part restored OS architecture.

To assess this more closely the OS length was quantified, revealing a strong difference between wt and *rd2* retina at P18 *in vivo* (wt P18: 13.88 μm ± 0.6 SEM, n = 3; *rd2* P18: 2.98 ± 0.5, n = 3; p = 0.002). At P19 *in vitro*, the *rd2* explants treated with 1.5 μM PJ34 exhibited significantly improved OS length, when compared to untreated *rd2* (untreated: 1.44 μm ± 0.1 SEM, n = 3; treated: 2.26 ± 0.1, n = 3; p = 0.007; Fig 3E). While in absolute terms this effect on OS length was small, in relative terms this corresponded to a more than 50% increase in OS length.

## Discussion

The enzymatic activity of PARP has been implicated with a variety of different diseases, including in cancer, neurodegeneration, and hereditary retinal degeneration [9,12,13]. In the present study, we show that the cellular localisation and temporal progression of PARP activity conforms to the progression of *rd2* photoreceptor degeneration, with the strongest PARP activity observed two days prior to the peak of cell death. Conversely, the inhibition of PARP delays *rd2* photoreceptor loss, causally linking PARP activity to the *rd2* degenerative mechanism. While previous works have shown the neuroprotective efficacy of PARP inhibition in rapidly degenerating *Pde6b*-mutant *rd1* retina [16,20], this is the first study showing that PARP inhibition is effective also in slowly degenerating mouse models suffering from *Prph2* mutations.

### PARP activity in photoreceptor degeneration

The enzymatic activity of PARP promotes DNA-repair [21,22] and is thus often associated with improved cell survival. There are at least 17 known PARP isoforms, which all use NAD^+^ to generate poly-ADP-ribose (PAR) polymers. These polymers are added either onto PARP itself (auto PARylation) or on other acceptor proteins. For instance, PARylation of DNA-binding histones [23] and the repulsive force generated by the negatively charged PAR polymers opens up the tightly packed chromatin structure, allowing dedicated repair enzymes to access the DNA [24]. After a successful DNA-repair, the PAR polymers are removed by poly-ADP-ribose glycohydrolase (PARG). The free PAR polymers generated by PARG activity may have signalling functions in their own right [25], while excessive PARP activity and consumption of NAD^+^ may indirectly cause ATP depletion, mitochondrial depolarization, and eventually cell death [26,27]. Therefore, strategies aimed at inhibiting PARP have become an important focus in recent therapeutic developments [12,13].

An excessive activation of PARP during photoreceptor cell death was first identified in the *rd1* mouse [20], an animal model suffering from a mutation in the *Pde6b* gene, which leads to rapid photoreceptor loss [28]. Later studies found a strong activation of PARP in dying photoreceptors in a variety of other animal models for hereditary retinal degeneration, including in P23H and S334ter rats [29] but also in the *rd2* mouse [9]. Even though, what was still unclear to this point was whether PARP activity was causally involved in the *rd2* degenerative process or whether it was merely an epiphenomenon caused by *rd2* cell death. Our present work illustrates the causal connection between excessive PARP activity and *rd2* photoreceptor degeneration, highlighting the possibility to use PARP inhibitors for the prevention of cell death in hereditary retinal degeneration caused by mutations in different genes.

### Neuroprotection with PARP inhibitors

Research into the therapeutic applications of PARP inhibitors initially focussed on their pro-apoptotic properties in cancer (reviewed in: [13,30], yet more recent studies also showed their neuroprotective qualities [31] including that of PJ34 [32]. Furthermore, several studies proposed PARP inhibitors for the treatment of hereditary retinal degeneration [9,20,33]. For instance, PJ34 was found to protect photoreceptors in retinal explant cultures derived from three different *Pde6a* mouse mutants [34]. Most recently, the PARP inhibitor olaparib showed a significant reduction of PARylation and cell death and, conversely, an increase in photoreceptor survival in *rd1* mouse retinal explant cultures *in vitro* and in *rd1* animals *in vivo* [16].

While in the rapidly degenerating *rd1* retina the peak of cell death and the peak of PARP activity could not be dissociated in time [20], in the far more slowly progressing *rd2* degeneration the largest amount of PARP activity was found to precede cell death by about two days. This provided a strong argument for PARP activity as a relatively early event during the *rd2* cell death process and may explain the efficacy of PARP inhibitors. Since in RP and LCA patient’s retinal degeneration usually progresses more slowly than in the mouse, this could also mean that the therapeutic window-of-opportunity in the human situation may by substantially larger. Remarkably, PARP inhibition appeared to partially restore outer segment morphology a finding that conforms to similar observations after *Prph2* gene therapy [19]. This could potentially be due to a PARP inhibition-induced over-expression of (defective) peripherin/rds protein or a compensation by other outer segment structural proteins such as prominin or GARP2 [1] and could be an interesting subject for future studies.

Here, it is worth mentioning that the retina and the eye as a self-contained organ offer a particularly attractive setting for further clinical developments: Local application of PARP inhibitors to the vitreous body [16] or into the Tenon capsule surrounding the eye [35] allows for a targeted compound delivery, essentially avoiding any concerns over potential systemic side-effects. What remains to be established for an *in vivo* application and potential clinical studies are suitable drug delivery systems that can combine with PARP inhibitors and enable long-term ocular delivery with the longest possible application intervals.

## Conclusions

Our study supports the idea of using PARP inhibitors for the treatment of hereditary retinal degeneration and diseases such as RP and LCA. Importantly, while previous studies had shown a protective effect of PARP inhibition in different rapidly degenerating *Pde6a* and *Pde6b* mutants [16,20,34], our work expands the group of RP genotypes potentially amenable to PARP inhibition treatment to also include slowly degenerating *Prph2* mutants. This highlights the possibility to use PARP inhibition as a general therapeutic strategy for the genetically extremely diverse group of hereditary retinal degenerations.
